# Assessing the impact of environmental exposures and *Cryptosporidium* infection in cattle on human incidence of cryptosporidiosis in Southwestern Ontario, Canada

**DOI:** 10.1371/journal.pone.0196573

**Published:** 2018-04-26

**Authors:** Gabrielle Brankston, Cyndi Boughen, Victoria Ng, David N. Fisman, Jan M. Sargeant, Amy L. Greer

**Affiliations:** 1 Department of Population Medicine, Ontario Veterinary College, University of Guelph, Guelph, Ontario, Canada; 2 School of Environmental Sciences, University of Guelph, Guelph, Ontario, Canada; 3 National Microbiology Laboratory, Public Health Agency of Canada, Guelph, Ontario, Canada; 4 Dalla Lana School of Public Health, University of Toronto, Toronto, Ontario, Canada; University of Minnesota, UNITED STATES

## Abstract

*Cryptosporidium* is a waterborne parasite that causes diarrheal disease in humans and in cattle. Risk factors for human illness include contact with surface water such as lakes and rivers, exposure to contaminated municipal drinking water, as well as zoonotic transmission from livestock and agriculture. The objectives of this study are twofold: 1) to describe the temporal distribution of cryptosporidiosis in Southwestern Ontario; and 2) to determine the distribution of human cryptosporidiosis, in relation to exposures such as cryptosporidium positive cattle farms, weather events, and hydrological factors. Seasonal trends in 214 bovine and 87 human cases were assessed using regression models that predicted monthly case counts in relation to observed monthly case counts. A case-crossover approach was used to evaluate acute associations between daily environmental exposures, such as weather, hydrology, the presence of *Cryptosporidium* positive cattle farms within the region, and the risk of human *Cryptosporidium* infection. Annual seasonality was found for both human cases and bovine cases with human cases peaking in mid-summer and bovine cases peaking in late winter to early spring. Bovine cases that occurred 21 days prior to human cases were associated with a three-fold increase in the odds of human case occurrence. At both 9 and 14 days prior to human case onset, the odds of a human case increased twofold per 10-degree Celsius increase in air temperature. These results provide a preliminary hypothesis for the zoonotic transmission of cryptosporidiosis from cattle to humans via the environment and suggest that the timing of environmental conditions in relation to case occurrence is biologically plausible.

## Introduction

Cryptosporidiosis is a human enteric illness that is reportable in the province of Ontario, Canada and is characterized by diarrhea, nausea, vomiting, and abdominal pain [1]. In Ontario, 1,048 cases of cryptosporidiosis were reported between 2007 and 2009, representing a mean annual incidence rate of 2.7 cases of illness per 100,000 population [2]. The highest incidence rates were reported in Southwestern Ontario [3]. Sources of *Cryptosporidium* infection in humans include contact with surface water such as lakes and rivers, as well as zoonotic transmission from livestock and agriculture [1,4]. Contaminated municipal drinking water is also a source of infection [1,4].

In humans, two species of *Cryptosporidium* (*C*. *hominis* and *C*. *parvum*) are responsible for the majority of *Cryptosporidium* infections [5]. *C*. *hominis* is specific to humans, while *C*. *parvum* is a zoonotic pathogen that has a wide range of hosts, including cattle [6]. *C*. *parvum* is responsible for approximately 85% of *Cryptosporidium* infections in pre-weaned calves [7,8]. In addition, nearly 100% of dairy calves become infected with *C*. *parvum* during their lifetime [6,7].

The seasonality of cryptosporidiosis has been well described. Human cryptosporidiosis cases occur seasonally, with a higher prevalence of disease during warmer and wetter months [9–11]. Specifically, within Ontario, Canada, human cryptosporidiosis peaks in the summer months [1–3]. Interestingly, a study from Prince Edward Island demonstrated a distinct spring peak in human shedding of *C*. *parvum* while *C*. *hominis* was most frequently detected between the months of July to September [5].

The manure of infected animals acts as a significant reservoir for *C*. *parvum* [12]. Infected calves can excrete up to 107 oocysts per gram of feces, leading to the shedding of millions of oocysts within the typical one- to two-week infection period [6,10,13,14]. The potential for environmental contamination leading to human exposure occurs when pathogen-loaded manure is spread onto fields as fertilizer, followed by high levels of precipitation [15,16]. Intense precipitation events cause varying degrees of surface water runoff, which can carry oocysts from farms into nearby watersheds, creating high pathogen loads and increasing opportunities for contact with humans [9,10,16]. Oocysts persist within cool moist environments, including manure, and remain viable for months in temperatures between 0°C and 20°C [17]. Once the pathogen enters the watershed, it becomes a human health hazard for those using surface water, such as rivers and lakes, for recreational purposes. In addition, oocysts may be resistant to conventional water treatment methods such as chlorine disinfection [18] and thus, may pose a risk for municipal drinking water.

In Ontario, contact with animals or with surface water are the main risk factors for human *Cryptosporidium* infections; 46.3% of 301 reported human cryptosporidiosis cases were linked to animal contact, and 38.5% were linked to surface water contact [4]. Local hydrological characteristics, such as water flow and level, may be important factors in the transmission of the pathogen, as oocysts can be distributed through watersheds. Indeed, the concentration of *Cryptosporidium* is significantly and positively correlated with water flow and turbidity during rainfall events [19].

While studies have demonstrated associations between each type of exposure (weather, hydrology, and veterinary health) and human illness, no known study has attempted to assess all of these factors concurrently to examine cryptosporidiosis at the human-animal-environment interface. This is the idea behind the One Health concept, a paradigm that regards human, animal and environmental health as inter-related, and seeks to develop linkages between human and veterinary realms [20].

Thus, the objectives of this study are twofold: 1) to describe the temporal distribution of human cryptosporidiosis in Southwestern Ontario, Canada; and 2) to determine the distribution of human cryptosporidiosis, in relation to exposures such as recent cases of bovine cryptosporidiosis, weather events, and hydrological factors.

## Materials and methods

This project was approved by the University of Guelph, Research Ethics Board (REB#15NV011).

### Study area

**Fig 1:**
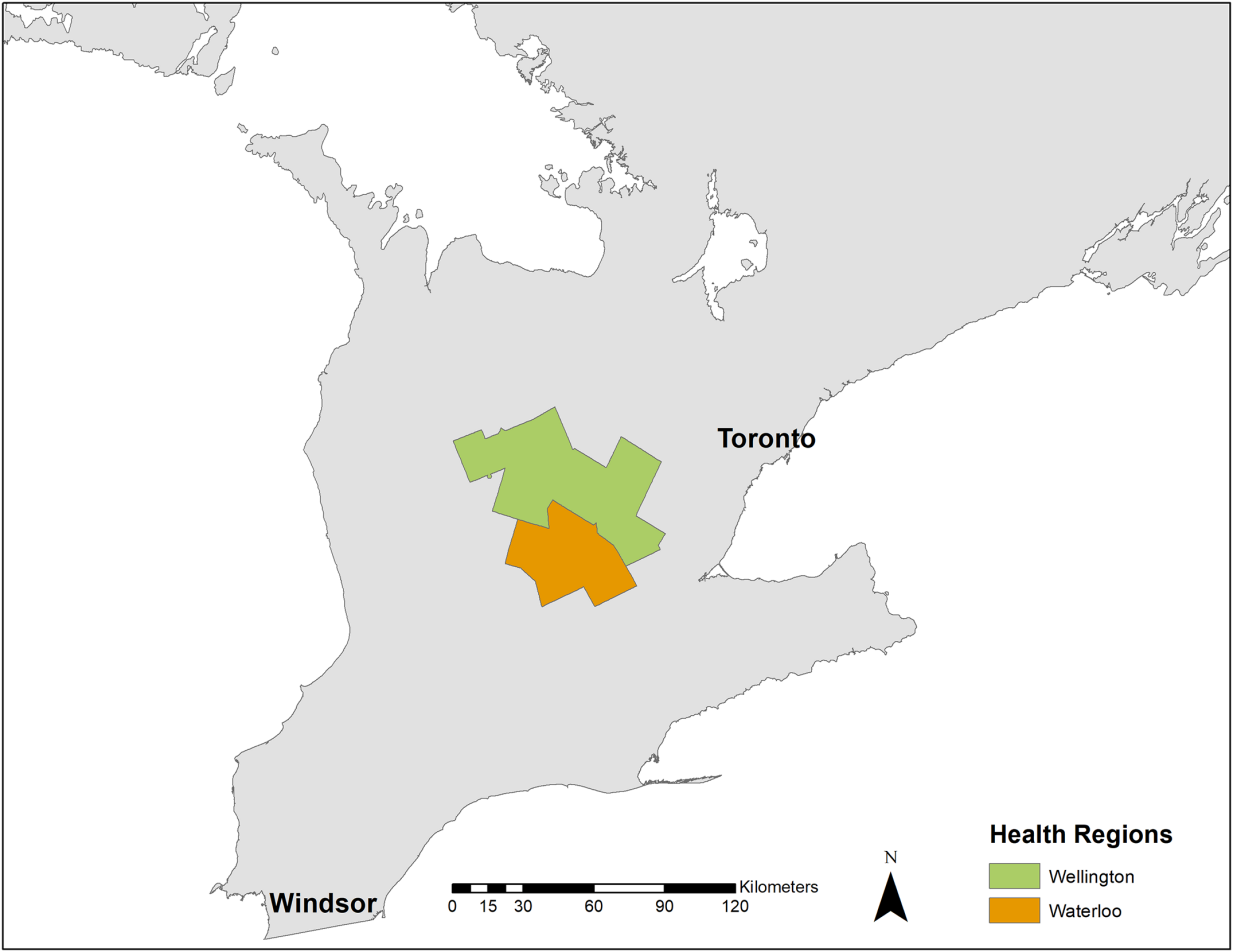
Map of Wellington and Waterloo counties (shaded) of Southwestern Ontario, Canada.

Wellington and Waterloo Counties of Southwestern Ontario, Canada (Fig 1) share the Grand River watershed which has a total drainage area of 6800km^2^ [16]. The majority of the watershed is surrounded by land used for agriculture and is home to 1294 cattle farms with a total cattle population of 233, 652 [21]. The majority of cattle farms within this region are dairy producers. The watershed is controlled by a system of seven dams and multiple reservoirs to mediate water flow in the region. The central area of the region is heavily urbanized with five major cities that include: Brantford, Cambridge, Guelph, Kitchener, and Waterloo [12]. These regions combined had a human population of 715,456 in 2011 [22].

### Human case data

Data on human cryptosporidiosis cases between January 1, 2009 and January 31, 2015 from Waterloo Region and Wellington, Dufferin, Guelph health unit areas were obtained from Public Health Ontario. Cryptosporidiosis is a reportable disease in Ontario and human case data represent cases reported to local public health agencies and then communicated to the Provincial health authority. Data were extracted from the integrated Public Health Information System (iPHIS), the electronic reporting system for reportable diseases in the province. A case was defined as an individual with laboratory-confirmed infection, with or without clinically compatible signs and symptoms, from an appropriate clinical specimen (i.e. stool). Cases associated with travel outside the province of Ontario during the relevant incubation period were excluded. A total of 87 cases were reported over the time period including 50 cases from Waterloo Region and 37 cases from Wellington, Dufferin, and Guelph. Daily case counts were aggregated to monthly case counts to assess seasonality and weekly case counts for univariate analyses.

### Cattle farm data

Data on bovine *Cryptosporidium* infections from farms located within the Waterloo Region and Wellington, Dufferin, Guelph health unit areas from January 1, 2008 to September 30, 2014 were obtained from the University of Guelph, Animal Health Laboratory (AHL). These data represent clinical samples submitted by Veterinarians to the AHL for diagnostic laboratory testing. While each submission to the lab could include clinical samples collected from multiple animals from a single farm, for our purposes, laboratory- confirmed “cases” were considered to represent farm-level positivity. Typically, veterinarians will not submit an individual sample from every symptomatic animal on a farm because a single positive laboratory result is enough to warrant treatment of groups within the herd. In this case, a farm was labelled “pathogen-positive” if any of the submitted clinical samples tested positive for Cryptosporidium. Within the region there were a total of 214 reported Cryptosporidium positive farms over the time period and cases were relatively evenly distributed throughout the two regions. For our purposes, cattle cases will refer to counts of laboratory-confirmed positive cattle farms. Daily positive, cattle farm counts were aggregated to monthly case counts to assess seasonality and weekly case counts for univariate analyses.

### Environmental exposure data

Hydrological data including daily flow (m^3^/s) and water level (m) from 18 Hydrometric Stations within the Grand River Watershed for the period from January 1, 2009 to December 31, 2014 were obtained from Environment Canada’s Water office website [23]. Daily water flow and level parameters for individual waterways were combined as average daily water flow and average water level for each region. Meteorological data including daily total precipitation (mm) and maximum air temperature (°C) for the period from January 1, 2009 to December 31, 2014 were obtained and averaged from two major Environment Canada active weather stations located in Fergus, ON (central Wellington County) and Waterloo, ON (central Waterloo County) [24]. Daily exposure data were aggregated into weekly exposure variables and one- to six-week lagged exposure variables.

### Statistical analyses

We evaluated the seasonal trends in disease occurrence using zero-inflated Poisson regression models that predicted monthly case counts in relation to observed monthly case counts. Seasonal trends in human cases were assessed during the time period between January 1, 2009 and January 31, 2015 (the full dataset that was available for human cases). The seasonal analysis for positive bovine farms was conducted for the time period between January 1, 2008 and September 30, 2014 (the full dataset that was available for bovine positive farms). The associations between weekly aggregated environmental exposures, weekly lagged environmental exposures (with one- to six-week lags), and weekly case counts were evaluated using both univariable and multivariable zero-inflated Poisson regression models for the time period between January 1, 2009 and September 30, 2014. This analysis utilized a shortened human dataset in order to match the available bovine farm dataset. Oscillatory seasonal smoothers, using sine and cosine terms, were included to control for non-specific seasonality of disease occurrence in such a way that environmental exposure variables identified as statistically significant would describe effects in excess of what would be expected based on seasonal oscillation alone [25]. Univariable models were used to assess unconditional associations between case counts and the exposure variables of interest. Exposure variables with p < 0.2 were considered to be of interest for a multivariable analysis [26].

Data were further analysed using a case-crossover approach to evaluate acute associations between human case occurrence and daily environmental exposures (including bovine cases) for the time period between January 1, 2009 and September 30, 2014. For this analysis, human and bovine case data along with environmental exposure data were no longer aggregated but were instead characterized by calendar day. This study design compares exposures identified during a defined hazard period directly prior to case onset with self-matched control periods. Exposures that increase the probability of case occurrence are expected to occur with greater frequency during the hazard period. This approach is used for evaluating the relationship between acute, short-term exposures and rare outcomes [27]. We used a time-stratified, 4:1 matched design in which four control periods were created and matched by day of week to each case onset day. To reduce bias we used random directionality for control period selection, such that control periods could follow, precede, or straddle the hazard period [28]. For this analysis, daily predictor variables, including 0 to 28 day lagged exposures were assessed for associations with human cases. Odds ratios for case occurrence were estimated using conditional logistic regression models. All statistical analyses were conducted using STATA 14.0 (STATA Corporation, College Station, TX).

## Results

### Seasonality

**Fig 2:**
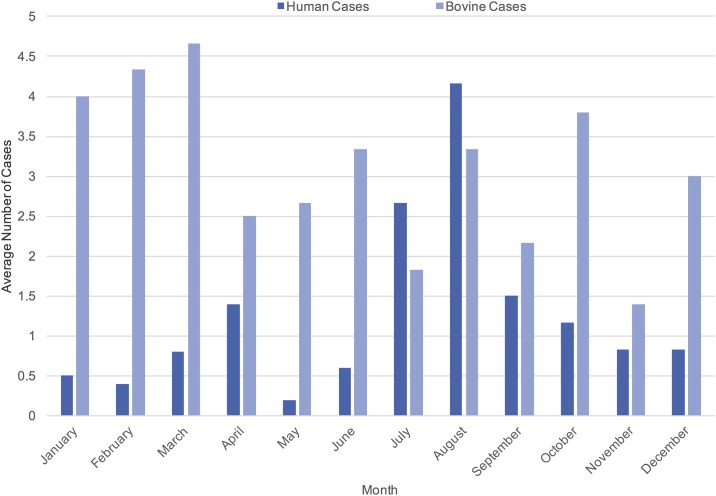
The average distribution of 214 bovine farm-level cases and 87 human cases of cryptosporidiosis, occurring in Waterloo/Wellington health regions 2009 to 2014, by month of onset.

We investigated 87 cases of human and 214 cases of cattle farm *Cryptosporidium* infection within the Wellington/Waterloo region. There was evidence for annual seasonality of both human cases (p < 0.001) and bovine cases (p = 0.027) with human cases peaking in mid-summer and bovine cases peaking in winter to early spring (Fig 2). There was no evidence for a yearly trend over the time period for either human or cattle cases.

### Zero-inflated Poisson regression

Controlling for seasonality, the univariable models showed no statistically significant associations between any of the exposure variables, including lagged variables, and human case counts. Thus, the multivariable model was not developed.

### Case-crossover analysis

In contrast with the results of the traditional regression analysis, the case-crossover approach demonstrated a number of statistically significant associations between human cases and environmental variables (Table 1). Cattle farm cases that occurred 21 days prior to human cases were associated with a three-fold increase in the odds of human case occurrence (Table 1). Maximum ambient temperature was positively associated with human cases 0, 9, and 14 days prior to human case onset. At both 9 and 14 days prior to human case onset, the odds of a human case increased by a factor of 1.07 per degree increase in air temperature (Table 1). Thus, during the same time period, a 10-degree increase in temperature would result in two-fold greater odds of human case onset (1.07^10^). Average water levels within the watershed were negatively associated with human cases 19–20 days prior to reports of human cases as each metre increase in water level resulted in a reduction in the odds of a human case by 74% to 81% (Table 1). Similarly, each 1 m^3^/s increase in average water flow was associated with a 7% reduction in the odds of human cases 28 days prior to human case onset. Precipitation was not associated with human cases of cryptosporidiosis.

**Table 1:** Statistically significant associations between environmental exposures and human cryptosporidiosis case onset identified using a case-crossover analysis.

Environmental Exposure	Odds Ratio	95% CI	p
**Number of positive cattle farms**			
** 3-day lagged**	0.14	0.02–0.99	0.05
** 21-day lagged**	3.04	1.34–6.90	0.01
** 28-day lagged**	0.11	0.01–0.80	0.03
**Maximum Ambient Temperature (°C)**			
** 0-day lagged**	1.08	1.02–1.15	0.01
** 9-day lagged**	1.07	1.01–1.13	0.03
** 14-day lagged**	1.07	1.00–1.14	0.04
**Average Water Flow (m^3^/s)**			
** 28-day lagged**	0.93	0.87–0.99	0.04
**Average Water Level (m)**			
** 19-day lagged**	0.26	0.07–0.94	0.04
** 20-day lagged**	0.19	0.05–0.71	0.01

## Discussion

Our results are in agreement with previous studies reporting seasonality of both cattle and human cryptosporidiosis [1,2,9,11,29]. The peak in cattle cases in the late winter/early spring prior to the summer peak in human cases is suggestive of zoonotic transmission. Destruction of oocysts has been demonstrated over winter in ambient temperatures fluctuating between -9°C and +9°C [30]. Similarly, oocysts become inactive in a 0°C environment [17]. It has been postulated that shear forces generated during freeze-thaw cycles destroys the parasites eliminating their ability to infect hosts [30]. This suggests that *Cryptosporidium* oocysts entering the environment from human infections would fail to persist over the typical winter in a Canadian climate, thereby likely eliminating humans as a potential reservoir for cattle infection. Conversely, oocysts may survive for extended periods in a 15°C environment [17]. This corresponds to typical springtime temperatures in Southwestern Ontario and would support the theory that late winter cattle cryptosporidiosis affects human case onset in the summer months.

Maximum ambient air temperature at 0, 9, and 14 days prior to human case onset was positively associated with human illness. These results are consistent with past research demonstrating that temperature of the current month, as well as temperature lagged by 1, 2 [31] and 3 months [32] were positively associated with human cryptosporidiosis. These results may seem counterintuitive given that the optimum conditions for oocyst survival and infectivity includes somewhat cooler temperatures [17]. However, this may be explained by people participating in outdoor recreation to a greater extent in warmer temperatures thereby increasing human exposure to pathogen-loaded surface waters.

Previous studies have shown that heavy rainfall is associated with an increased incidence of waterborne illness. A two-fold increase in the odds of a waterborne disease outbreak was found for rainfall events greater than the 93rd percentile compared with rainfall events less than the 93rd percentile [33]. Similarly, a significant increase in weekly cryptosporidiosis rates has been demonstrated for accumulated rainfall above the 75^th^ percentile for the previous week [34]. In theory, heavy rainfall should flush manure and any associated pathogens into surface water sources. Indeed, the concentration of *Cryptosporidium spp*. has been shown to be positively correlated with water flow and turbidity during rainfall events [19] however, not all species of *Cryptosporidium* are associated with human illness.

The results from the present study found no association between rainfall and the incidence of human cryptosporidiosis. These results are consistent with a small body of evidence finding either no association [31] or a negative association between rainfall and cryptosporidiosis incidence [35]. Similarly, a study from England and Wales reported that 20% of waterborne disease outbreaks in the twentieth century were associated with extended periods of low rainfall compared with 10% of outbreaks associated with periods of heavy rainfall [36]. Correspondingly, the current study demonstrated that higher average water levels 19–20 days prior to human case onset are associated with lower odds of human illness. It is possible that periods of low rainfall can lead to increased pathogen concentration in environmental water sources resulting in an increased likelihood of infection. By the same token, increased water levels in waterways may dilute the concentration of oocysts thereby reducing the likelihood of infection for those in contact with these water sources.

While hydrological factors such as stream flow may impact the movement of pathogens at the soil level, this study found a negative association between average water flow and the incidence of human illness 28 days prior to case onset. Thus, higher water flow was associated with lower odds of human illness. Similarly, it has been demonstrated that the detection of high densities of *Cryptosporidium* oocysts has been associated with low stream flow [37]. When water flow conditions are insufficient to dilute or flush pathogens from waterways, it could be hypothesized that pathogens may become concentrated. Conversely, high water flow may dilute or flush pathogens from waterways.

It is likely that the manipulation of the watershed via the dam and reservoir system that exists within this region confounded the effect of rainfall and watershed variables on human illness in the present study. Reservoirs in the Grand River watershed fill during the spring runoff and reach peak levels at the beginning of June. Some of the water is stored in order to reduce downstream flood peaks during this time. As natural flows decline in the summer and fall, the water stored in the reservoirs is slowly released to add to the natural flows in the river system. For example, in 2012, water flow augmentation via the reservoir system peaked in July and August [38]. The slow release of water from the reservoir system during periods of dry weather creates a more consistent water level and flow throughout the season, resulting in an artificial assessment of the effects of rainfall and hydrology variables on the risk of human illness within this region.

Overall, the results from this study provide a preliminary hypothesis for the zoonotic transmission of cryptosporidiosis from cattle to humans via the environment, suggesting that the timing of environmental conditions in relation to case occurrence is biologically plausible. Human cryptosporidiosis has an average incubation period of seven days [39]. This dynamic may explain the relationship with environmental conditions 9 to 14 days prior to cases occurring as well as the presence of positive cattle farms occurring 21 days prior to human cases. The lag times in this study provided sufficient time for the environment to be contaminated, for humans to be exposed to the contaminated environment via recreational use of surface waters, for an appropriate incubation period in humans, and for people to seek medical care and submit a stool sample. While we cannot assume a causal association between temperature, positive cattle farms, and human cryptosporidiosis risk, we believe that our results provide an indication of specific, high-risk time periods when human infections are more likely to occur. Identification of these higher risk periods can be communicated to the public in order to reduce the likelihood of exposure via recreational waters.

Two fundamentally different methods of analysis were used in this study and produced contrasting results. The regression analysis evaluated aggregated case counts in relation to weekly averaged measures of environmental exposures, while the case-crossover methodology evaluated the acute effect of daily environmental exposures on the occurrence of human cases. The difference in results between the two analytical methods may represent an example of “ecological fallacy” from the use of weekly aggregated case and exposure data. An ecological fallacy occurs when an analysis of aggregated data is used to draw conclusions about an individual’s risk of illness [40]. This emphasizes the importance of supplementing traditional epidemiological analysis with complementary methodologies when examining the effects of acute environmental exposures on health outcomes.

### Limitations

Relatively few human cases of cryptosporidiosis were reported in Waterloo and Wellington, Dufferin, Guelph health regions during the study period. While cryptosporidiosis is a reportable illness in Ontario, underreporting of acute gastrointestinal illness in Ontario is quite common [41]. Treatment for mild gastrointestinal illness tends to focus on supportive therapy including rest and rehydration and people may self-treat based on experience rather than seek medical care. As with human gastrointestinal illness, cattle farm data likely represents only a portion of all farms experiencing disease in their herds. Cattle *Cryptosporidium* infection is quite common and farmers are likely to treat their livestock based on experience, without help from a veterinarian or lab confirmed diagnosis, which may limit our statistical power.

Misclassification in terms of human case location may have biased the results. For location purposes in public health surveillance, human cases are counted in the health unit of primary residence. Thus, illness acquired elsewhere will still be counted as a case for the health unit in which the case resides. Similarly, the location data for cattle cases are typically based on veterinary location rather than farm premise location. Veterinary premises may not be located within the same health region as the farms they service. Therefore, we cannot be certain of the exact location of infected farms.

Molecular epidemiology was not available for either human or cattle cases. Similarly, environmental sampling data were not available. While the results provide evidence for the linkages between human, animal, and environment, we cannot be certain that all three entities were infected or contaminated with the same species of *Cryptosporidium*. In a study such as this one, where we have evaluated multiple environmental and livestock exposures it may be important to consider the role of multiplicity and consider how many exposures we might expect to be positive by chance alone.

Future research should include fieldwork that includes environmental sampling and genetic identification of *Cryptosporidium* species. Additional environmental exposures, including UV index, should also be assessed as ultraviolet rays have been shown to reduce the length of time that the *Cryptosporidium* oocyst is able to remain viable [42]. Future studies should include regions without a dam system to control for the confounding effect of such a system. The addition of these factors may provide further insight into the transmission dynamics of cryptosporidiosis as well as reveal additional conditions under which an increased or decreased risk of infection may occur.

In conclusion, our results identify specific conditions within the environment that are associated with an increased risk of cryptosporidiosis in humans in the southwestern region of Ontario, Canada. These findings lead to a biologically plausible explanation for the observed seasonality of the disease and identify environmental conditions putting people at an increased risk for illness. Ongoing research in this area will enable us to build a more comprehensive understanding of the complexities of *Cryptosporidium* as a zoonotic pathogen in order to better prevent and control pathogen spread amongst cattle, people and within the environment.
